# Human Alveolar Echinococcosis, Croatia

**DOI:** 10.3201/eid2602.181826

**Published:** 2020-02

**Authors:** Davorka Dušek, Adriana Vince, Ivan Kurelac, Neven Papić, Klaudija Višković, Peter Deplazes, Relja Beck

**Affiliations:** University Hospital for Infectious Diseases, Zagreb, Croatia (D. Dušek, A. Vince, I. Kurelac, N. Papić, K. Višković);; University of Zagreb School of Medicine, Zagreb (D. Dušek, A. Vince, N. Papić);; University of Zurich, Zurich, Switzerland (P. Deplazes); Croatian Veterinary Institute, Zagreb (R. Beck)

**Keywords:** Echinococcus multilocularis, human, Croatia, emerging diseases, parasites, parasitic diseases, tapeworms, echinococcosis, zoonotic diseases, zoonoses

## Abstract

Alveolar echinococcosis is a parasitic disease caused by the tapeworm larval stage of *Echinococcus multilocularis*. This zoonotic disease has not been known to occur in Croatia. We report a confirmed case of human alveolar echinococcosis in a patient in Croatia who had never visited a known *E. multilocularis*–endemic area.

A 63-year-old male patient was sent to the University Hospital for Infectious Diseases in Zagreb, Croatia, in September 2017 for treatment of cystic liver lesions and pleural effusion. The patient had grown up and still lived in a rural area in Vukovar (45°21′N, 18°59′E/45.35°N, 18.99°E), where he worked for a waste management company. He spent free time in the woods picking mushrooms.

Before his referral, in November 2014, the patient underwent kidney ultrasonography, which also detected cystic formations in his liver. A subsequent multislice computed tomography (MSCT) scan in a regional hospital revealed an oval heterogeneous zone in the liver measuring 11 × 8 cm with irregular postcontrast enhancement and an enlarged right suprarenal gland that could not be distinguished from the outer wall of the inferior vena cava. His laboratory results, including tumor markers, were within reference ranges, except for a slightly elevated alkaline phosphatase level. 

In March 2015, histopathology of liver tissue excluded malignant disease. The patient tested positive for *Echinococcus* sp. by enzyme immunoassay and Western blotting, but cystic echinococcosis was excluded on the basis of radiologic findings. In June 2015, a second liver biopsy revealed necrotic cells. In November 2015, another MSCT revealed multiple new nodular lesions in the right and left lungs. Thorax and lung biopsies revealed necrotic material.

In 2016, the patient had a bronchoscopy, which showed no malignancy. He had a third diagnostic liver biopsy in October 2016, which revealed acellular, eosinophilic, periodic acid-Schiff–positive histopathology, and morphology that suggested echinococcosis. However, the patient was not seen by an infectious disease physician, nor did he receive treatment for parasites. 

An examination in August 2017 showed that his lung nodules had progressed, and a right-sided pleural effusion had enlarged. One month later, the patient’s condition worsened. He had a fever (39°C), dyspnea, cough, and sharp pain in the right hemithorax. He was referred to the hospital, where we conducted a cystic lesion biopsy, which indicated suppurative inflammation, but a culture was negative for bacteria. The patient’s pleural effusion had eosinophilic exudate, which was negative for bacteria and fungi. The patient had eosinophilia with an average eosinophil count of 2,600 × 10^9^ cells/L. We analyzed his prior MSCT and magnetic resonance scan results and noted the patient had experienced slow progression of alveolar echinococcosis. The liver lesion had grown from 11 × 8 cm to 13 × 12 × 12 cm during the previous 2.5 years, and the parasite had infiltrated his lungs and right adrenal gland (Figure).

**Figure:**
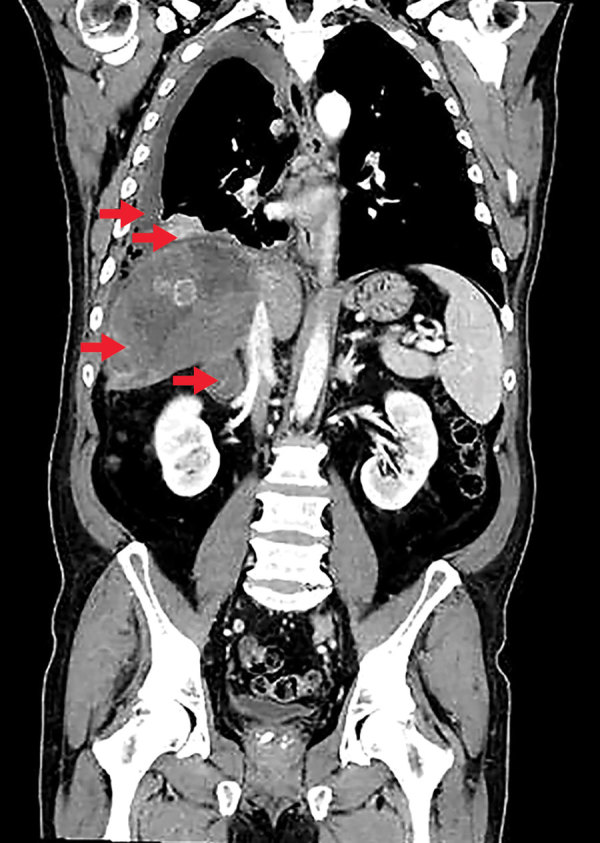
Computed tomography scan of a patient diagnosed with alveolar echinococcosis, Croatia. Arrows indicate right pleural effusion, lung lesions, an enlarged right adrenal gland, and a 13 × 12 × 12 cm lesion in the liver caused by *Echinococcus multilocaris*.

We extracted DNA from pleural exudate and formalin-fixed, paraffin-embedded liver biopsy tissue by using the QIAamp DNA Mini QIAcube system (QIAGEN, https://www.qiagen.com). We subjected duplicate samples to PCR and sequenced a 200-bp region in the mitochondrial gene *nad1* (*1*) and a 395-bp region of the 12S rRNA gene (*2*). All sequences were identical to isolates of *E. multilocularis* tapeworms from Europe found in GenBank (accession nos. MG755265, MG755266).

Serologic tests using various antigens (*3*) performed at the University of Zurich (Zurich, Switzerland) further supported the diagnosis of alveolar echinococcosis. We obtained strongly positive results with genus-specific ELISAs conducted with somatic protoscoleces or cyst fluid from *E. granulosus* sensu stricto and with ELISAs highly specific for *E. multilocularis* tapeworm based on the recombinant Em18 antigen or affinity-purified native EmG11 antigen. Serum analysis conducted by using the Anti-Echinococcus EUROLINE-IgG Western Blot system (EUROIMMUN, https://www.euroimmun.com) showed positive results for p7, p28, and Em18.

The patient refused surgery and was treated with oral albendazole (400 mg 2×/d) and intravenous piperacillin/tazobactam (4.5 g 4×/d) for 6 weeks. At discharge, we recommended continued albendazole until clinical and radiologic follow-up 6 months later.

No previous human cases of alveolar echinococcosis have been reported in Croatia, but the *E. multilocularis* tapeworm is endemic in central and eastern Europe and in the Baltic states (*4*). This patient’s infection is surprising because he lived >60 years in eastern Croatia with no international travel. The closest reported autochthonous human cases were in southwestern Hungary (*5*). 

*E. multilocularis* tapeworms have been reported in foxes in western and central Croatia (*6*) and likely is in eastern areas, such as Vukovar, because it was found in 17.9% of foxes and 14.3% of golden jackals in the region of Serbia directly across the Danube River from Vukovar (*7*). Since 2013, rabies vaccination has increased in Croatia, which might give the fox population an opportunity to expand and increase transmission of *E. multilocularis* tapeworms to humans, as noted in Switzerland (*8*).

Correct diagnosis for this patient took 2.5 years because radiologic findings were inconsistent with cystic echinococcosis and clinicians assumed that was the only type of human echinococcosis in Croatia (*9*). This case highlights the need for clinicians to include alveolar echinococcosis in differential diagnosis of liver lesions. Imaging provides the first-line approach to such a diagnosis and serology provides strong complementary support. Our case also highlights the usefulness of considering pleural effusion and analyzing archival biopsies to retrospectively diagnose alveolar echinococcosis.
